# Innate Immunity as an Executor of the Programmed Death of Individual Organisms for the Benefit of the Entire Population

**DOI:** 10.3390/ijms222413480

**Published:** 2021-12-15

**Authors:** Boris V. Chernyak, Konstantin G. Lyamzaev, Armen Y. Mulkidjanian

**Affiliations:** 1 Belozersky Institute of Physico-Chemical Biology, Lomonosov Moscow State University, 119992 Moscow, Russia; lyamzaev@gmail.com; 2 School of Bioengineering and Bioinformatics, Lomonosov Moscow State University, 119992 Moscow, Russia; 3 Department of Physics, Osnabrueck University, D-49069 Osnabrueck, Germany

**Keywords:** pathogen-associated molecular patterns (PAMPs), damage-associated molecular patterns (DAMPs), mitochondrially-targeted antioxidants, inflammation, inflammasome, programmed death, phenoptosis, COVID-19

## Abstract

In humans, over-activation of innate immunity in response to viral or bacterial infections often causes severe illness and death. Furthermore, similar mechanisms related to innate immunity can cause pathogenesis and death in sepsis, massive trauma (including surgery and burns), ischemia/reperfusion, some toxic lesions, and viral infections including COVID-19. Based on the reviewed observations, we suggest that such severe outcomes may be manifestations of a controlled suicidal strategy protecting the entire population from the spread of pathogens and from dangerous pathologies rather than an aberrant hyperstimulation of defense responses. We argue that innate immunity may be involved in the implementation of an altruistic programmed death of an organism aimed at increasing the well-being of the whole community. We discuss possible ways to suppress this atavistic program by interfering with innate immunity and suggest that combating this program should be a major goal of future medicine.

## 1. Introduction

The concept of altruistic programmed death of whole organisms was proposed by Vladimir Skulachev more than two decades ago [1,2]. It was based on a suggestion that the death of individuals, if programmed, may be a subject to Darwinian selection and contribute to inclusive fitness. This suggestion expanded the original idea of inclusive fitness, also known as kin selection, which initially was about promoting reproduction and survival of genetic relatives only via social behavior [3]. In addition to this concept, Skulachev suggested that Darwinian selection may have shaped the mechanisms “of clearing a kin community of organisms or a population from individuals who have become unwholesome for this community”. He suggested to call this type of kin selection “phenoptosis”. Skulachev suggested that “the septic shock and stress-induced ischemic diseases of brain and heart” could be considered as the examples of phenoptosis [1]. At the same time, “slow phenoptosis” was proposed as an equivalent term for programmed aging [1,2,4].

The concept of phenoptosis has received support in experimental [5] and computer studies of *Caenorhabditis elegans* [6,7]. A cold shock followed by rewarming induced expression of genes of proteases, which promoted the programmed organismic death [5]. In the model of a clonal population of *C. elegans* that subsists on spatially limited food sources, simulations have shown that shorter lifespan can increase the colony fitness [6,7]. Presumably, phenoptosis, by reducing the futile food consumption by elderly or stressed worms leads to adaptive benefits for the colony. This concept is also fully consistent with the recently published results of mathematical modeling [8]. The authors demonstrated that in a population of short-lived individuals, the spread of infection is limited, and the pathogen clearance is more efficient than in population in which members live longer.

For diverse types of programmed cell death (PCD), such as apoptosis, necroptosis, and pyroptosis, specific execution mechanisms were identified [9]. Therefore, to categorize phenoptosis as a specific bona fide program, a specific biochemical execution mechanism(s) has to be identified as well. Here we review diverse evidence that the execution of innate immune system mechanisms can lead to animal death and discuss some possible means to interrupt potentially lethal innate immune system programs.

## 2. Innate Immunity, Pathogen-Associated Molecular Patterns, and Pattern Recognition Receptors

The basic principle of innate immunity, as formulated by C. A. Janeway Jr [10], states that diverse invading pathogens are immediately recognized by molecules that are broadly shared by pathogens, such as glycans, N-formylated peptides, flagellin, and nucleic acids, but are distinguishable from host molecules. These molecules are collectively referred to as pathogen-associated molecular patterns (PAMPs). Some PAMPs are shared by viruses and diverse groups of microorganisms, including non-pathogenic ones.

PAMPs are recognized by pattern recognition receptors (PRRs). The first of the discovered PRRs are Toll-like receptors (TLR), of which there are at least 10 in humans [11,12]. Later several further classes of PRRs were discovered including C-type lectin receptors (CLRs), retinoic acid-inducible gene-I (RIG-I)-like receptors, NOD (nucleotide-binding oligomerization domain)-like receptors (NLRs), and cytosolic DNA sensors [13,14].

TLRs are located in the plasma membrane or in the membranes of intracellular compartments (endoplasmic reticulum, endosomes, lysosomes), recognizing a wide variety of substances of microbial and viral origin [11,15].

CLRs make a large group of transmembrane or soluble receptors expressed primarily by myeloid cells. They recognize carbohydrates (glycans) on the surface of bacteria, fungi, helminths, and viruses [16].

The RIG-I-like receptors are intracellular viral RNA receptors. This family includes RIG-I that recognizes RIG-I circular RNA and short (≤300 bp) dsRNAs, MDA5 that senses long dsRNAs, and LGP2 that binds dsRNA and regulates signaling by RIG-I and MDA5 [17].

The NLR family has 22 members in humans, which are cytosolic sensors expressed in various immune and non-immune cells [18]. The NOD1 and NOD2 receptors recognize the degradation products of bacterial peptidoglycan, in particular muramyl dipeptide, which is characteristic of numerous pathogenic bacteria. The NLRP1, NLRP3, and NLRC4 receptors, in response to pro-inflammatory stimuli, form inflammasomes, large multiprotein complexes in which caspase-1 is activated to convert the precursors of interleukin 1β (pro-IL-1β) and interleukin 18 (pro-IL-18) into their active forms and to stimulate their release [18,19,20]. In addition to NLRs, the inflammasome formation can be induced by the interferon-inducible protein AIM2, the cytosolic DNA sensor [21].

The main PRR that recognizes the cytosolic DNA is the cyclic guanosine monophosphate adenosine monophosphate synthase (cGAS) that, by using STING (cGAS stimulator of interferon genes), stimulates the production of type-I interferon (IFN-I) [22]. Several other soluble receptors for cytosolic DNA and RNA have recently been identified [21]. All these sensors recognize DNA that can emerge during the life cycle of intracellular pathogens including viruses, bacteria, and parasites.

## 3. Damage Associated Molecular Patterns (DAMPs) and Their Receptors

**Figure 1:**
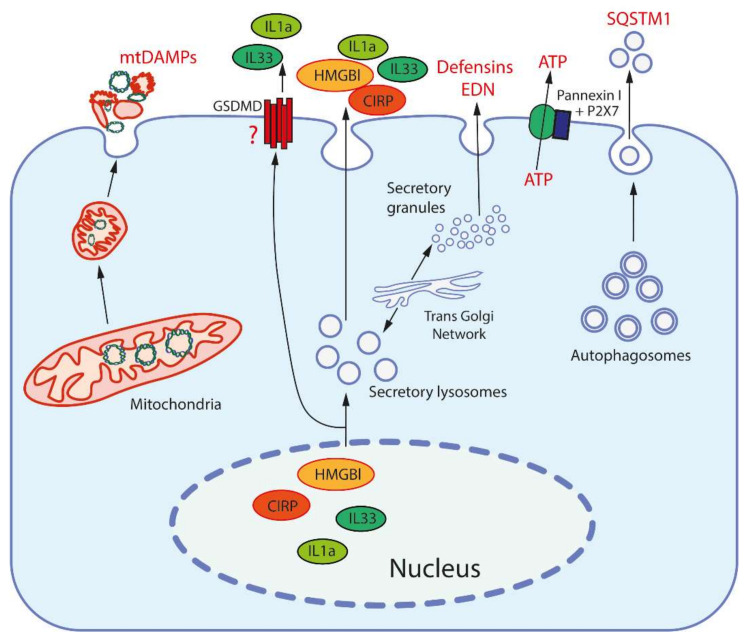
Release mechanisms for some DAMPs. High-mobility group protein B1 (HMGB1) and cold-inducible RNA-binding protein (CIRP) are released from the nucleus after modification to be entrapped and secreted by secretory lysosomes (which are produced by the trans-Golgi network). Interleukins 1a and 33 (IL1a, IL33) are also secreted by secretory lysosomes, but may be also released through the pores formed by gasdermin D (GSDMD). Mitochondria, after fragmentation, can be extruded from cells by an unknown mechanism(s). Secretory granules, as produced by the trans-Golgi network, release defensins and eosinophil-derived neurotoxin (EDN) by exocytosis. ATP is released through the pore formed by pannexin I complexed with the purinergic receptor P2X7. Sequestosome-1 (SQSTM1/h62) is an autophagosome receptor that can be released during secretory autophagy.

In addition to PAMPs, the same TLRs, as well as some other PRRs, recognize damage-associated molecular patterns (DAMPs), which are endogenous molecules released from damaged cells, see [23,24] and Figure 1. Various types of DAMPs and their role in inflammation were extensively reviewed [25,26].

The concept of DAMPs stems from the “danger model” of immunity by P. Matzinger [23]. In contrast to the traditional “self versus non-self” recognition principle, she proposed that the immune system recognizes danger signals regardless of their origin. Initially, this hypothesis concerned adaptive immunity [23], but later the same principle was applied to innate immunity [24].

DAMPs can directly initiate at least two forms of PCD (pyroptosis and NETosis), which are involved in the pathogenesis of sepsis, as well as many other diseases associated with aseptic inflammation. Pyroptosis is a form of cell death that is accompanied by activation of a pore-forming protein gasdermin D by inflammatory caspases [27]. NETosis (where NET is deciphered as neutrophil extracellular traps) is a specific form of cell death that is characterized by the release of weblike DNA structures decorated with histones and antimicrobial proteins by neutrophils [28]. Cytokines produced in response to DAMP can also initiate other forms of PCD, such as apoptosis and necroptosis, which also contribute to various pathologies.

Today, a large number of DAMPs of various chemical nature and origins have been identified (see Table 1), and this list is expanding rapidly. The most important and well characterized DAMPs, namely the high-mobility group protein B1 (HMGB1) and cold-inducible RNA-binding protein (CIRP), function as chaperons in the nucleus [29,30]. Two interleukins of the IL-1 family, IL-1α and IL-33, are nuclear proteins capable of regulating expression. Outside the cell, they are recognized not by PRRs, but by the receptors of the IL1R family (IL-R1 and ST2, respectively), which transmit signals using the same MyD88 adapter as some TLRs [31]. Some other cytokines, such as thymic stromal lymphopoietin (TSLP) and IL-25, are also considered DAMPs although their intracellular functions have not been defined [31]. Expression of all these DAMPs is stimulated by various stresses and by mediators of inflammation.

Cytosolic DAMPs, such as S100 proteins that regulate Ca^2+^ signaling [31], actin, a component of cytoskeleton [32], peptidyl prolyl isomerase cyclophilin A [33], antioxidant protein peroxiredoxin 1 [34], and heat shock proteins (HSPs) [35] are among the most abundant proteins. Oxidized hemoglobin and heme, as released from red blood cells upon hemolysis, also act as DAMPs [36].

Secretory granules are another important source of DAMPs; these are antimicrobial peptides (defensins and LL37) [37], eosinophil-derived neurotoxin (EDN) [38], and granulisin, which accumulates in the granules of human cytotoxic T lymphocytes and natural killer cells [39].

Molecules of ATP [40] and uric acid (UA) [41] are the most important low molecular weight metabolites involved in the activation of inflammation upon release from the cell. In addition, various components of the extracellular matrix (ECM), including proteoglycans, soluble glycoproteins, low molecular weight hyaluronan, and heparan sulfate, are capable of acting as DAMPs [42,43].

Mitochondria are regarded as an important source of DAMPs (mtDAMPs) primarily due to their bacterial origin. Mitochondria-synthesized proteins are N-formylated, and mitochondrial DNA (mtDNA) contains insufficiently methylated CpG regions, similarly to the corresponding bacterial components which are recognized as PAMPs. One example of mtDAMPs in common with some bacteria is cytochrome *c* [44]. In contrast, the transcription factor A (TFAM) is an example of a mtDAMPs that appears not to have a bacterial counterpart [43]. Almost the entire list of known DAMPs can be released from tumors and contribute to immunogenic cell death (ICD) associated with an adaptive immune response [45]. Some of the DAMPs (not all), including those that are predominantly released from tumors, are listed in Table 1.

DAMPs can be released from cells via active and passive pathways (Figure 1). Passive release requires the rupture of the plasma membrane and occurs during pyroptosis, necroptosis, or necrosis. Both nuclear DNA and histones can be released from leucocytes in form of decondensed chromatin forming extracellular traps (ETs; NETs in the case of neutrophils). Interestingly, some DAMPs (HMGB1, CIRP) can induce release of NETs, which leads to the formation of an amplification loop [46].

Active release of nuclear DAMPs, such as HMGB1 and CIRP, requires their posttranslational modification in order to leave the nucleus, and subsequently occurs through exocytosis of secretory lysosomes [29,30]. The same exocytotic mechanism participates in active release of cytosolic and mitochondrial DAMPs. ATP can be released not only from damaged cells, but also through the channel formed by the P2X purinoreceptor 7 (P2X7 receptor) together with pannexins [40]. Uric acid excretion is largely regulated by urate transporters in the renal tubules [41]. In general, it can be assumed that the release of the most important DAMPs may be mediated by specific mechanisms in living cells (Figure 1).

Initially, it was thought that DAMPs are only recognized by pattern recognition receptors (PPRs), but later the receptors for advanced glycation end products (RAGE), triggering receptor expressed on myeloid cells 1 (TREM-1), CD91 and purinergic receptors were also shown to participate in the DAMP recognition [25,26]. It has recently been reported that sequestosome 1 (SQSTM1/p62), a known selective autophagy receptor released by macrophages and monocytes, is recognized by the insulin receptor (INSR) to stimulate inflammation [53]. In addition, formyl peptide receptor 1 (FPR1), is a G protein-coupled receptor that recognizes N-formylmethionine-containing peptides [54].

Some DAMPs can be recognized without release from the cell. MtDNA, released from mitochondria into cytosol and oxidized, can stimulate the NLRP3 inflammasome activity [55]. Another intracellular DAMP is cardiolipin, a four-tail phospholipid which is located exclusively in the inner mitochondrial membrane. Under stress, cardiolipin is externalized on the mitochondrial surface and stimulates the assembly of the NLRP3 inflammasome [49].

**Figure 2:**
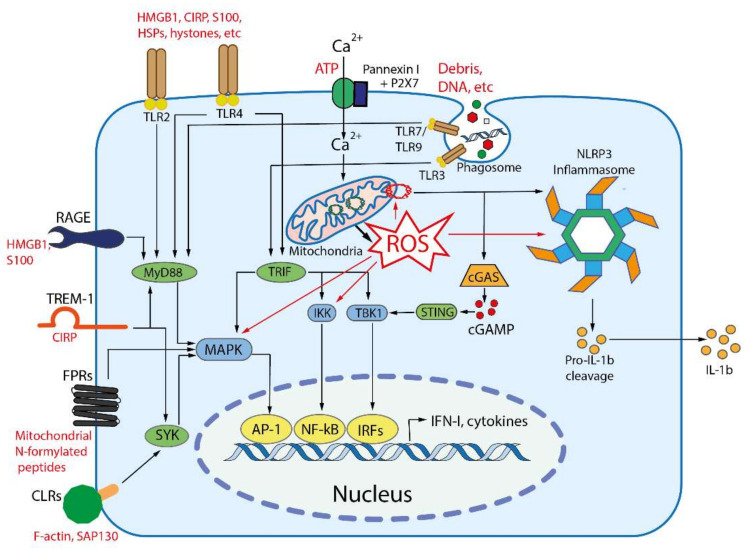
Main DAMP-sensing receptors and signaling. DAMPs can be recognized by membrane receptors, such as Toll- like receptors (TLRs), receptor for advanced glycation end products (RAGE), triggering receptor expressed on myeloid cells 1 (TREM-1), formyl peptide receptors (FPRs), C-type lectin receptors (CLRs), purinergic receptor P2X7, as well as by cytoplasmic receptors such as cyclic GMP-AMP synthase (cGAS) and NLR family pyrin domain containing 3 (NLRP3) inflammasome. Endogenous nuclear and mitochondrial DNA released into the cytoplasm activate cGAS, which produces cyclic GMP-AMP (cGAMP) that binds to stimulator of interferon genes (STING). Activated STING stimulates the kinases TBK1 and IKK, leading to the expression of type-I interferons (IFN-I) and inflammatory cytokines. TLR2 and TLR4 can be activated by a variety of extracellular DAMPs, whereas TLRs 3, 7, and 9 are activated by phagocytosed nucleic acids in endosomes. TLR-dependent signaling is mediated by the adaptor proteins: myeloid differentiation primary-response 88 (MyD88), and TIR-domain-containing adaptor inducing IFNβ (TRIF). The both adaptors activate mitogen-activated protein kinases (MAPKs) and IκB kinase (IKK), leading to activation of the transcription factors activator protein 1 (AP-1) and nuclear factor κB (NFκB), which stimulate the expression of inflammatory cytokines. TRIF also activates TANK binding kinase 1 (TBK1), which activates the interferon regulatory factors (IRFs) leading to the expression of type I interferon (IFN-I). RAGEs, which recognize HMGB1, S100 and some other DAMPs, also activate MyD88. TREM-1, which recognizes CIRP, activates spleen tyrosine kinase (SYK), that further stimulates MyD88, MAPKs and NFκB. Several C-type lectin receptors (CLRs) recognize F-actin and SAP130 and also activate SYK. Formyl peptide receptors (FPRs) are G-protein coupled receptors that recognize mitochondrial N-formylated peptides and stimulate several signaling pathways (including MAPKs) to activate neutrophils. P2X7 is ionotropic receptor that opens Ca^2+^ channel in response to extracellular ATP. An increase in the concentration of Ca^2+^ in cytoplasm modulates various signaling pathways and stimulates the production of reactive oxygen species (ROS) in mitochondria. Elevated ROS levels are critical for the activation of MAPKs, NFκB and the NLRP3 inflammasome. ROS-oxidized mitochondrial DNA is recognized by NLRP3.

Signal transduction from DAMP-sensing receptors uses some of signaling modules that are activated by elevated cytosolic levels of Ca^2+^ and reactive oxygen species (ROS). The main outcomes of DAMP-dependent signaling include NFκB and inflammasomes activation, followed by the production of inflammatory cytokines and chemokines, activation of mitogen-activated protein kinases (MAPK), and stimulation of interferon signaling (Figure 2), see [26] for a comprehensive review.

In sum, DAMPs, as well as PAMPs, initiate an innate immune response, which involves the release of cytokines and other inflammatory mediators, activation of some specific defense mechanisms (for example, the production of extracellular traps), and induction of certain types of programmed cell death. Such a combination of organismic reactions is commonly called inflammation, which has been recently defined as “the innate immune response to harmful stimuli such as pathogens, injury, and tissue malfunction” [56]. This definition covers both acute (local or systemic) and chronic inflammatory processes.

## 4. PAMP- and DAMP-Dependent Pathologies

Innate immunity is an important defense system against pathogens. This system includes not only the inflammatory response, but also effective mechanisms for resolving inflammation and stimulating tissue repair and regeneration. However, concurrently with the protective effects of PAMPs and DAMPs recognition, activation of innate immunity is associated with adverse outcomes in many pathological conditions.

### 4.1. Bacterial Infections

Bacterial PAMPs induce an acute systemic inflammatory reaction in various severe infections. For example, the immune reaction to the cholera pathogen *Vibrio cholerae* infection persists for up to 30 days after the end of the infection [57]. Moreover, dehydration in cholera is suppressed by antibodies against TLR4 and inhibitors of inflammatory signaling pathways [58]. A similar picture is observed with meningitis caused by various bacterial pathogens [59].

Various DAMPs, such as HMGB1, CIRP, HSP70, hyaluronan, and mtDAMPs contribute to the pathogenesis of bacterial pneumonia and its more severe form, acute respiratory distress syndrome (ARDS) [60]. The potential role of mtDAMPs in ARDS has been demonstrated in the pioneering work of Hauser and colleagues [61]. In this study, intravenous injection of isolated mitochondria was shown to cause an increase in IL-6 and neutrophil infiltration with subsequent lung injury. Later it was confirmed that mitochondrial formylated peptides and mtDNA play a crucial role in inflammatory response, neutrophilia, and damage to epithelial cells of the airways and microvascular endothelial cell in the lungs during ARDS [62].

### 4.2. Viral Infections

DAMPs release and inflammation were found to accompany various viral infections. Analysis of bronchoalveolar lavage fluid revealed the presence of S100A9 and HDM1 proteins in the airways of mice infected with influenza A virus (IAV) [63,64]. Moreover, injection of neutralizing monoclonal antibodies significantly reduced the mortality of IAV-infected mice, but did not affect virus propagation in the lungs [63,64]. TLR4−/−knockout mice were found to be resistant to both H5N1 [65] and mouse-adapted lethal H1N1 influenza [66].

Human respiratory syncytial virus (HRSV) is a leading cause of serious pediatric respiratory infections and can also cause severe mortality in the elderly and immunocompromised individuals. HRSV promotes the HMGB1 release from airway epithelial cells [67], and high HMGB1 levels were detected in nasopharyngeal samples of children with acute HRSV infection [68]. In a genetically susceptible mice infected with the mouse homolog of HRSV, the secretion of HMGB1 was suppressed by pharmacological inhibitors of necroptosis. These inhibitors prevented progression of disease in this model [68].

In the dengue virus infection, leukocyte and platelet-mediated inflammation caused by viral PAMPs and DAMPs (primarily histones) led to thrombosis and vascular leakage, hemorrhage, and organ failure [69].

### 4.3. COVID-19

The death toll from the coronavirus infection SARS-CoV2 (COVID-19) exceeds 5 million worldwide. It is becoming generally accepted that the pathogenesis of COVID-19 has two phases: an early phase, when viral infection predominates, and a later phase, when immune responses determine a severe course of the disease. In support of this scheme, a very low viral lung burden was found in samples from patients who died from COVID-19 during the initial outbreak in Wuhan, China [70], which indicates that the active infection was not the immediate cause of death. It is still unknown why, in some cases, the immune response gets out of control whereas the infection is mostly stopped by antiviral systems. Cain and Sidlowski [71] hypothesized that the release of DAMPs, which leads to a hyperinflammatory state (including a “cytokine storm”), is critical to the pathogenesis of severe COVID-19.

In support of this hypothesis, levels of various DAMPs have been shown to increase during severe COVID-19 and correlate with poor prognosis. High serum levels of S100A8/A9 and HMGB1 found in COVID-19 patients upon admission to hospital have been correlated with worse clinical outcomes [72,73]. S100A8/A9 (calprotectin) has been shown to be a more sensitive marker than HMGB1 and various cytokines, but S100A8/A9 and HMGB1 levels were similarly elevated in critically ill patients [72]. The increase in S100A8/A9 during COVID-19 correlates with changes in monocyte and neutrophil subsets associated with the stimulation of emergency myelopoiesis [74,75]. Other DAMPs elevated during COVID-19 and correlated with poor outcome include extracellular nuclear DNA [76], mitochondrial DNA [77], and IL-33 [78].

### 4.4. Sepsis

**Figure 3:**
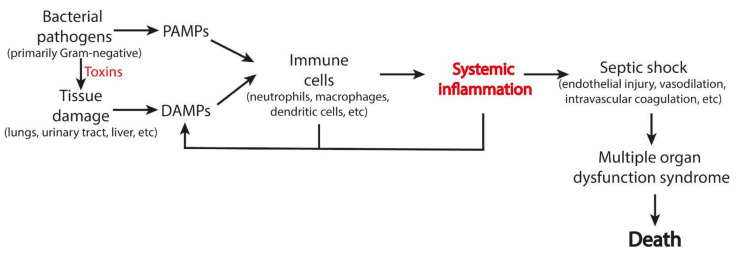
Scheme of the induction of phenoptosis in bacterial sepsis. This scheme can also be applied to pathologies associated with aseptic inflammation.

Sepsis is a fatal condition that accounts for up to 50% of deaths in intensive care units (ICU) worldwide. Septic shock arises from an excessive immune response to infection and was originally associated solely with a response to PAMPs. Later, however, the release of DAMPs was recognized as the critical event in mortality in sepsis. Clinically, the severity of sepsis correlates with the level of some DAMPs, such as HMGB1 [79], CIRP [80], histones [47], HSP70 [81], and circulating plasma DNA [82]. Mitochondrial DAMPs [83] and extracellular ATP [84] were also suggested to contribute to sepsis mortality. DAMPs contribute to the pathogenesis of sepsis by stimulating cytokine production, NET release, pyroptosis, immunothrombosis, etc. (Figure 3).

### 4.5. Sterile Inflammation

Systemic sterile inflammation is an inevitable consequence of massive trauma (incl. surgery), burns, hemorrhagic shock, ischemia/reperfusion injury, and some toxic insults [85]. The symptoms of systemic sterile inflammation generally overlap with those of sepsis, and often (especially in pediatrics) these conditions are very difficult to discriminate. A number of DAMPs, including HMGB1, CIRP, histones, HSP, S100, ATP, and mtDAMPs, have been shown to play a key role in sterile inflammation.

### 4.6. Neuroinflammation and Neurodegeneration

Neuroinflammation plays an important role in the pathogenesis of stroke, traumatic brain injury, neurodegenerative diseases (Alzheimer’s disease (AD), Parkinson’s disease, Huntington’s disease), amyotrophic lateral sclerosis (ALS), and multiple sclerosis (MS). Microglia, astrocytes, and oligodendrocytes constitute the local innate immune system in the brain, and DAMP-dependent activation of these cells underlies neuroinflammation. Immune cells from the bloodstream can enter the brain due to disruption of the blood–brain barrier after trauma or outbreaks of MS. TLRs, RAGE, and P2X7 expressed in neurons and glial cells contribute to the pathogenesis of stroke, trauma, ALS, and MS [86].

The etiological role of inflammation in neurodegenerative diseases is still a matter of debate. It was shown that amyloid β, one of the recognized markers of AD, interacts with TLR4 and plays the role of DAMP [48]. It is assumed that other known DAMPs (HMGB1, S100, HSP), as well as mitochondrial DAMPs, contribute to the pathogenesis of AD [87].

It is noteworthy that severe neurological disorders often accompany systemic inflammation. Sterile systemic inflammation caused by surgery [88] or cardiac arrest [89] is associated with encephalopathy, which manifests itself in conditions ranging from delirium to coma. Encephalopathy is a common complication of the acute liver failure and pancreatitis [90], as well as the acute injury of kidneys [91] and lungs [92] with poor outcomes. The pathogenesis of encephalopathy includes microglia activation, inflammation, cerebral ischemia, neurotransmitter imbalances, and blood–brain barrier dysfunction. Encephalopathy followed by long-term cognitive problems is a well-known complication of sepsis [93]. A nationwide study in Denmark showed a significant increase in the risk of suicide within 2 years after sepsis was cured [94]. Importantly, studies in both animals [95] and humans [96] have shown that microglial activation and inflammation during sepsis is not associated with any known cerebral infection. DAMP-dependent signaling has been shown to be responsible for encephalopathy caused by hepatic failure [90] and cerebral ischemia [97,98]. Importantly, in severe respiratory viral infections such as COVID-19, the encephalopathy, delirium, and long-term cognitive impairment are correlated with systemic inflammation [99].

## 5. Suppression of Inflammation by Targeting Mitochondria

Mitochondria not only supply a significant share of DAMPs (see Table 1), but, more importantly, control the main signaling pathways involved in the activation of innate immunity and inflammation [100,101]. Mitochondrial ROS (mtROS) are responsible for the activation of NADPH oxidase in neutrophils [102], as well as the release of NETs [103], important components of antimicrobial defense and, at the same time, important pathogenic factors. MtROS are critical for cell damage and control some forms of PCD, which, in turn, leads to the release of DAMPs [104].

One of the key players in the mitochondria-mediated immune response is the four-tail phospholipid cardiolipin that is specifically localized in the mitochondrial membranes. The four tails of cardiolipin are usually polyunsaturated to enable their packing in the lipid bilayer; therefore, cardiolipin is particularly prone to oxidation by mtROS. Oxidized cardiolipin molecules, because of the peroxide groups, strive to leave the inner mitochondrial membrane and can appear on the surface of the outer mitochondrial bilayer where they trigger different regulatory cascades, see [105,106,107,108,109]. In addition, the oxidation of cardiolipin affects its ability to prevent proton leakage across the inner mitochondrial membrane, which causes a drop in the membrane potential and activates a further set of regulatory pathways [108,110,111].

Oxidation of cardiolipin by mtROS is known to trigger different types of PCD which could be accompanied by the release of mitochondrial DAMPs (Table 1) in the blood flow [112,113,114]. Earlier it was shown that mtROS are involved in the activation of NLRP3 inflammasome in macrophages [115] and thus in production of inflammatory cytokines. More recently it was demonstrated that the activation of NLRP3 inflammasome was triggered by the externalization of cardiolipin [49,116]. Mitochondrial ROS production and cardiolipin-mediated oxidative stress contribute to the pathogenesis of septic encephalopathy [117], ischemia/reperfusion injury [118], neurodegeneration [119], autoimmune diseases [120], and many other pathologies associated with the activation of innate immunity. Specifically, the mitochondria-driven inflammatory response is believed to play a key role in the genesis of multiple-organ failure observed in sepsis [104,121,122].

Not surprisingly, mitochondria-targeted antioxidants, which specifically protect cardiolipin molecules from oxidation [105,106,107,108,109], prevent many of the above mitochondria-mediated adverse effects, see [9] and [123] for comprehensive reviews. One of the first mitochondria-targeted antioxidants, MitoQ, a conjugate of a ubiquinone headpiece with a lipophilic cation triphenylphosphonium (TPP^+^) decreased markers of acute hepatic and renal dysfunction [124] as well as cardiomyopathy [125] in a rat model of endotoxemia. The mitochondria-targeted antioxidants developed by Skulachev and colleagues (SkQs, conjugates of a plastoquinone headpiece with TPP^+^), suppressed the manifestations of brain damage, myocardial infarction, and kidney injury induced by ischemia [126]. The MitoQ [127] and SkQs [128] significantly dampened the symptoms of the Alzheimer’s disease in animal models.

The beneficial effects were not limited to the quinone-based mitochondria-targeted antioxidants. The antioxidants that used conjugates of a stable aminoxyl radical 2,2,6,6-tetramethylpiperidin-1-yl)oxyl (TEMPO) as an antioxidant moiety, being targeted to mitochondria, also suppressed inflammatory response both in vitro, in the cell models, and in vivo, in the mice septic models [129,130,131]. For one of such conjugates, the hemigramicidin-TEMPO conjugate XJB-5-131, the ability to almost completely protect cardiolipin of oxidation in a traumatic brain injury model was demonstrated [106].

In a completely different line of evidence, it was shown that long omega-3 polyunsaturated fatty acids (PUFAs) suppress the activation of NLRP3 inflammasome [132]. In addition, it has been shown that these fatty acids can be incorporated into cardiolipin and increase the average length of its tails [133]. Longer hydrophobic tails should decrease the probability of externalization of cardiolipin molecules which is necessary for activation of NLRP3 [49,116]. Thus, long omega-3 PUFAs appear to specifically inhibit mitochondria-mediated inflammation. This effect, at least in part, explains the well-established anti-inflammatory action of omega-3 PUFAs, primarily eicosapentaenoic acid and docosahexaenoic acid in various human inflammatory diseases, including diabetes, atherosclerosis, asthma, and arthritis [134,135].

Hence, innate immunity-mediated inflammation can be suppressed by targeting its signal pathways which pass through mitochondria.

## 6. Biomarkers of the All-Cause Death

Several years ago, Fischer and colleagues published a seminal paper on a search for biomarkers that correlated with all-cause mortality [136]. Using NMR, the authors looked at 106 different biomarkers in blood plasma samples that were stored for many years and compared their data with the available health records. The authors found that the levels of four biomarkers—plasma albumin, alpha-1-acid glycoprotein, very-low-density lipoprotein (VLDL) particle size, and citrate—appeared to accurately predict the short-term risk of death. The same four biomarkers were identified for Finnish and Estonian sets of blood samples that were analyzed separately.

Plasma albumin and alpha-1-acid glycoprotein were categorized by the authors as inflammation markers, whereas the VLDL, generally known as “bad cholesterol”, was considered as a marker of triglyceride state. The authors did not explain how high levels of citrate in blood could promote all-cause deaths, but provided a reference to the observation that elevated citrate levels were predictive of death from sepsis in hospital settings [137]. Later, however, O’Neill and colleagues argued that elevation of cytosolic citrate level in macrophages, which is coupled to the citrate level in the blood, can be considered an inflammatory signal [138,139]. Furthermore, the accumulation of citrate is expected in response to elevated ROS production because the citrate-processing aconitase is the most ROS-sensitive enzyme in the Krebs cycle [140]. Hence, the elevated level of citrate in the blood may also correlate with inflammation and/or mitochondrial damage.

More recently, the analysis of Fischer and colleagues was repeated with a much larger set of plasma and serum samples [141]. The four initially identified markers were confirmed; in addition, several new types of markers were identified. Specifically, the all-cause deaths were shown to correlate with low levels of polyunsaturated fatty acids. Polyunsaturated fatty acids were categorized by the authors of the study as inhibitors of inflammation, in agreement with literature data [134,142,143,144,145,146,147]. In a separate study, Harris have shown that the risk of all-cause death is specifically decreased by very long chain 3-omega fatty acids, namely, eicosapentaenoic, docosapentaenoic, and docosahexaenoic acids. Other polyunsaturated fatty acids did not show compatible beneficial effect [148].

Hence, the studies of the biomarkers of all-cause mortality indicate that most of them correlate either with inflammation or with the damage to the cell or mitochondria.

## 7. Innate Immunity-Mediated Phenoptosis as a Common Cause of Human Mortality

In the previous sections we have emphasized that manifestations of innate immunity mechanisms in many pathological conditions led to death even when the pathological agents (viruses or bacteria) cannot cause death per se. We also showed that such fatalities could be prevented by interfering, in diverse ways, with the specific innate immunity regulatory pathways controlled by mitochondria. In addition, it was demonstrated that the probability of death outcome (from all causes) correlates with the presence of certain blood biomarkers most of which are related to inflammation.

Up to now, the above-described fatalities were attributed to the imperfection of the innate immunity system causing its overreaction. However, the respective mortality rates are very high, up to 50% in the case of sepsis, even in the best intensive care units around the world. High mortality is also inherent in various pathologies associated with local inflammation (ischemic and toxic lesions of the brain, heart, kidneys, liver, etc.), and in some pathologies where inflammation is not clearly expressed, but DAMP plays an important role in pathogenesis (including aforementioned neurodegenerative diseases). Such extremely high, seemingly wanton mortality must have an evolutionary cause. More likely is that (i) the high mortality, as caused by sepsis, sterile systemic inflammation, and some described severe infections, is a manifestation of phenoptosis that aims at cleansing the population of unwanted individuals, and (ii) innate immunity reactions are in a similar way involved in all the above-described cases because phenoptosis uses them as an executive mechanism. Involvement of the same innate immunity pathways, under certain circumstances leading to death, explains the similarity of symptoms in the generalized, pathogen-induced inflammation including sepsis and in sterile types of inflammation.

Cancers also fit into this framework. As early as in 1994, Sommer suggested that cancer “has a biological role in that it mediates evolutionary selection for a constant rate of germline mutation” (quoted from [149]) [1]. Cancer can be one of the most important mediators of negative selection, not only in the case of mutations (germline, as well as somatic), as discussed by Sommer [149], but also in pathologies related to systemic inflammation. Although it was repeatedly shown that inflammation can provoke cancer, inflammation plays a dual role in cancer [150]. On the one (dark) side, DAMPs released from tumors during progression or as a result of therapy can directly promote invasion and metastasis by interacting with PRRs expressed on tumor cells [151,152]. Moreover, DAMP-induced chronic inflammation in the tumor microenvironment attracts immunosuppressive cells such as M2 macrophages, myeloid suppressor cells and regulatory T cells, helping tumors escape immunosurveillance [153]. On the other (bright) side, DAMPs contribute to immunogenic tumor cell death caused by conventional therapy or modern cancer immunotherapy [45].

Generally, the existence of specific phenoptotic programs is indicated by the observations that such non-trivial symptoms as thrombosis and vascular leakage, hemorrhage, and organ failure were observed in relation to quite different causes, such as influenza, COVID-19, and some other respiratory viral infections, as well as in bacterial pneumonia, cholera and so on [69,154,155].

The existence of a distinct program gives a hope of preventing phenoptosis by interfering with the checkpoints of the program. Involvement of mitochondria as one of such checkpoints explains the aforementioned therapeutic effects of mitochondria-targeted antioxidants and very long chain 3-omega fatty acids in a variety of severe pathologies. It is noteworthy, that the impact of mitochondria-targeted antioxidants and very long chain 3-omega fatty acids is not just beneficial, but life-saving. Specifically, SkQ1-type antioxidants were the first to show a decrease in mortality in the kidney ischemia/reperfusion model [126], in models of pyelonephritis [156] and neonatal endotoxemia [157], as well as in a murine model of systemic inflammation induced by intravenous injection of TNF [158].

Identification of mitochondria as a phenoptosis checkpoint is also supported by a recent observation that high plasma level of very long omega-3 fatty acids lowered the risk for a fatal outcome in case of COVID-19 [159]. Most likely, omega-3 fatty acids prevented the formation of the NLRP3 inflammasome, which was activated in response to SARS-CoV2 and was found to be abundant in various tissues of postmortem patients upon autopsy [160].

In addition, the possible interruption of phenoptotic programs at the recognition stage of PAMPs or DAMPs could be a useful strategy to combat various life-threatening diseases. If so, the phenoptosis execution may be prevented by diminishing the level of certain DAMPs. And indeed, genetic knockouts, neutralizing antibodies, and pharmacological agents that inhibit DAMPs or prevent the activation of DAMP receptors such as TLRs, RAGE, NLRP3 inflammasome, and P2X7 ATP receptor, significantly attenuate the course and reduce mortality in animal models of systemic sterile inflammation [161]. The serum S100A8/A9 and HMGB1 levels were correlated an increased risk of lethal thrombosis [162]. Paquinimod, a specific inhibitor of S100A8/A9, reduced the number of aberrant neutrophils, eliminated lung damage, and protected mice in a lethal model of murine coronavirus infection [75]. Neutralizing antibodies against DAMPs significantly improved animal survival in models of endotoxemia and sepsis [46]. Small molecule inhibitors that prevent the secretion of HMGB1 and serum protein haptoglobin, which binds to extracellular HMGB1, have also been shown to have protective effects [163]. The important role of CIRP has been confirmed by the findings that CIRP−/− knockout mice are resistant to CLP sepsis [30]. In support of the role of extracellular histones in sepsis, several negatively charged molecules, including heparin, chondroitin sulfate, and high molecular weight hyaluronan, which can bind to histones of NETs were shown to protect against sepsis [164]. P2X receptor blockade or treatment with apyrase, that removes extracellular ATP, protects mice from lethal endotoxemia [46].

One more checkpoint might be on the level of T-cells. Kim et al. who studied the cytokine storm in lymphocyte-depleted mice showed that T cells are necessary and sufficient to temper the overreaction of the innate immune system [165,166].

In sum, the available data indicate that the program of inflammation-mediated phenoptosis could be interrupted in several checkpoints.

## 8. Phenoptosis and Aging

The close link between the immune system and aging has been confirmed in genetic studies of longevity in *Drosophila melanogaster*. In three independent studies [167,168,169], the long-term (up to 35 years) selection of flies for longevity and subsequent analysis of genomes revealed important changes in the genes of innate immunity. Moreover, conditional knockdown of the Toll-receptor in adulthood using in vivo RNA interference (RNAi) significantly increased lifespan of the flies [169]. In other studies, RNAi-mediated suppression of inflammatory NF-κB signaling in glia [170] or of the secretion of antimicrobial peptides [171] led to an increase of *D. melanogaster* lifespan by more than 60%.

An experiment similar to that described above with *D. melanogaster* was realized in nature during the evolution of bats. Bats are known to contain more viral pathogens than any other mammalian species, being a kind of viral reservoir. The unique mechanisms of immune tolerance allow bats to coexist peacefully with various pathogenic viruses, including coronaviruses [172,173,174]. Bats have a strong interferon response to RNA viruses, but reduced sensitivity to viral DNA. In part, this reflects the absence (in various bat species) of the PYHIN gene family, which includes the cytoplasmic DNA sensors AIM2 and IFI16 capable of activating the inflammasome and interferon pathways. In addition, bats have a mutated STING that is incapable of cGAS-dependent activation critical for IFN activation. Another DNA-sensitive receptor TLR9 is also mutated, and its activation by CpG-containing oligonucleotides is reduced. Moreover, the NLRP3 inflammasome, which is a key sensor for various pathogens, is suppressed at several levels, including mutations in caspase-1 [175]. In some bat species, additional mechanisms of immune tolerance have been described, such as upregulation of anti-inflammatory IL-10 expression and downregulation of TNF expression [172]. Bats are the only flying mammals, and it is likely that immune tolerance evolves in them as an adaptation to active metabolism and muscle work, which are accompanied by an increase in body temperature and the release of DAMP [174]. High body temperature also can partially inactivate viral pathogens.

It is important to note that bats live significantly longer than other mammals of the same size. Typically, bats have a maximum lifespan of about 20 years, while some species of bats (such as Brandt’s bat and little brown bat) have a maximum lifespan of 30–40 years [172]. It is tempting to speculate that the development of immune tolerance in bats suppressed phenoptosis, resulting in an increase in their lifespan.

If the high body temperature that occurs during flight helps bats to partially suppress the innate immune system and coexist with viruses, a similar strategy would be expected in long-lived birds. Certain bird species, such as some albatrosses (family *Diomedeidae*) and some parrots (family *Psittacidae*), are known for their extreme longevity. Although we are not aware of systematic studies of innate immunity in long-lived birds, a single study reported very low level of TLR diversity, which might indicate a certain suppression of innate immunity [176].

Recent studies on an extremely long-lived (without age-related increased risk of death) naked mole rat (*Heterocephalus glaber*), show that its innate immune system lacks natural killer cells, but shows an elevated (compared to other rodents) myeloid to lymphoid ratio and increased production of pro-inflammatory cytokines in macrophages [177,178]. The authors suggest that naked mole rats living in underground tunnel systems, with little risk of viral infection, need a good antibacterial protection because of their crowded lifestyle. These data indicate that the naked mole rat, faced with a bacterial threat, implements a strategy somewhat opposite to that of bats, which have developed means to control viral infections. It should be noted that in both cases, the suppression of components of innate immunity—albeit different components—correlates with increased longevity. We believe that further studies of innate immunity in long-lived species will further shed light on its role in aging and phenoptosis.

Notably, the possibility of switching innate immunity components off might be constrained by their involvement in other cellular functions critical for development or survival. For example, Toll receptors are involved in the development of Drosophila [179]. The important DAMP HMGB1 is also involved in development, and only its conditional knockouts are viable [29]. Mice with knockout of CIRP (another important DAMP) while viable and resistant to hemorrhagic shock and sepsis [30], have delayed wound healing [180], which may point on a suppressed stress response.

Unfortunately, unlike bats and naked mole rats, humans are immune intolerant enough to cause potentially lethal innate immune system reactions, as discussed in previous sections. One may wonder why hominids did not alleviate phenoptosis during their evolution by suppressing some components of their innate immune system, as bats and naked mole rats did? All the more so because the survival of older and more experienced members capable of transferring knowledge is of particular value in human communities where knowledge and skills are passed on through intergenerational communication rather than inheritance.

The high immune intolerance in humans may have several causes. First, the components of the innate immune system are highly conservative, as discussed in the following Section 9, so that hundreds of thousands of years of knowledge and skills transmission through communication may not have been sufficient to modify the innate immune system. Second, humans until recently have been vulnerable to bacterial and viral infections (there is no medication against the latter even now), so phenoptosis by the innate immune system has played a role in protecting human communities from extinction.

At this point we have a conundrum between the need to keep the innate immunity combat-ready and the need for relatively long lifespan for knowledge transfer.

Evolution seems to have found a solution to this conundrum by using a different mechanism, not related to the innate immune system, to prolong human life. As first suggested by Bolk [181], the development of human individuals exhibits features of neoteny (juvenilization), namely the presence of childhood features in adult organisms, which should delay ageing and extend lifespan. A detailed review of this feature in humans has recently been published [182]. As a result, modern humans have both a longer lifespan and a vigilant innate immune system.

Aging of humans is known to be accompanied by low-grade inflammation, as manifested by consistently elevated levels of inflammatory cytokines, especially interleukin-6 (IL-6) and tumor necrosis factor-α (TNF-α); these factors could promote diverse pathologic states such as diabetes and cancer [183,184,185]. A considerable amount of evidence supports the hypothesis of Salvioli and colleagues that low-grade inflammation not only accompanies old age, but is one of the main factors of aging [186,187]. Furthermore, Franceschi and colleagues emphasized the relation between low-grade inflammation and aging by coining the term “inflammaging” [188].

Hence, innate immunity mechanisms can work both in a fast, acute mode, as in the case of severe infections or sepsis, and in slow or even chronic modes, as in the case of low-grade inflammation.

Concurrently, Skulachev suggested that aging can be described as a slow phenoptosis and mtROS play a key role in senile pathologies and in “healthy aging” [1,2]. In support of the Skulachev’s conjecture of aging as slow phenoptosis, mitochondria-targeted antioxidants (SkQs) have been shown to significantly increase lifespan in various animal models [189]. These observations suggest that activation of innate immunity, associated with the production of mtROS, is an executive mechanism of both acute phenoptosis and aging.

These multiple parallels between the aging-related features of innate immunity and phenoptosis indicate a profound connection between the two and support our assumption that innate immunity systems are executors of phenoptotic programs.

## 9. Co-Evolution of Immunity and Phenoptosis

While the very existence of phenoptosis is still debatable for multicellular organisms, its presence in unicellular organisms is undeniable because the phenoptosis of such organisms is equivalent to their demise through PCD. Indeed, starting from prokaryotic organisms, host-pathogen interactions are the main driving force behind the co-evolution of immunity and phenoptosis; in the course of this co-evolution, bacteria and archaea evolved diverse abortive infection systems [190,191]. Some of them are based on toxin-antitoxin (TA) modules. These modules consist of a stable toxin that can potentially kill a cell and an antitoxin that is inactivated by phage infection. For example, the *Thermus thermophilus* genome contains 12 putative TA loci [192]. More recently, the TA system has been described in hyperthermophilic archaeon *Pyrococcus yayanosii* [193].

The restriction-modification systems are another important part of prokaryotic “innate immunity” [194]. At least some of these systems possess suicidal defense mechanisms that destroy host DNA upon phage infection [191].

The best characterized “abortive infection system” of *Escherichia coli* is based on RexA/RexB module, which stops cell division to protect against spread of λ-phage infection [194]. In the experiment, two *E. coli* strains carrying the λ prophage with or without Rex were mixed. The strain without Rex has been shown to outcompete the Rex-positive strain in conventional well-mixed culture. However, in a “structured medium” (it is enough to add 1.5% agar to the medium to limit the spread of bacteria) the Rex-positive strain gained an advantage [190]. These results are in accord with mathematical modeling, which showed that individual suicide can be an adaptive host defense strategy in an infected population that satisfies two conditions: (i) a spatially structured environment that limits free mixing within the population, and (ii) an extremely dangerous pathogen with a high transmission rate [195]. Important to note that these two conditions are fulfilled in most communities throughout the tree of life, and they are easy to simulate experimentally.

Furthermore, the systems of prokaryotic adaptive immunity CRISPR-Cas were shown to be involved in a suicidal activity. CRISPR (Clustered Regularly Interspaced Short Palindromic Repeats grouped at regular intervals) make in prokaryotic genomes a collection of sequences derived from the genomes of phages that have previously infected the host [191]. These systems help to recognize the DNA of similar phages upon infection and destroy it using Cas endonucleases, which proceeds under strict control to prevent the erroneous destruction of the self-DNA. Still, the CRISPR-associated suicidal activities were described, which could be either related or unrelated to nuclease activity. Furthermore, one of the rare CRISPR-Cas systems (type VI) bypasses the control mechanisms and induces cell suicide in a “panic” response to any DNA invasion [191]. Genomic studies have shown that those genomic loci that encode the CRISPR-Cas systems are co-localized with those loci that encode the toxin-antitoxin modules and restriction-modification systems (two mechanisms of suicidal defense) [191], which implies an evolutionary and functional relationship between the immune systems and the system of altruistic suicide in prokaryotes.

Both innate (abortive infection) and adaptive (CRISPR-Cas) systems are widespread in bacteria and archaea, including extremophiles [192,193,196]. Studies of CRISPR ecology and evolution have revealed a high diversity between different extremophile species [197]. Importantly, Koonin and his colleagues found that the loss of a family of defense genes occurs about three-times more often than amplification [198], which might be a consequence of adaptive suppression of the prokaryotic immune systems in different lineages (by analogy with bats).

The eukaryotic innate immunity, based on recognition of PAMPs by TLRs, emerged very early in the phyla Porifera (sponges), Cnidaria (corals, *Hydra*, jellyfish), and Mollusca [199]. Not much is known about the possible recognition of DAMPs in these species. The main purpose of PAMPs’ and DAMPs’ sensing in all species from primitive animals to humans is to attract immune cells to sites of infection or injury to fight pathogens and facilitate tissue repair and regeneration. In mammals, DAMP-dependent signaling is involved in the resolution of inflammation by stimulating phagocytes to efficiently cleanse cellular debris and dead cells [200] and to produce anti-inflammatory cytokines such as IL-10 [201]. Further contributions to the mechanisms of tissue repair include direct or indirect stimulation of hematopoiesis, proliferation and differentiation of epithelial cells and fibroblasts, angiogenesis, and remodeling of the extracellular matrix. DAMP-dependent TLR signaling is required for liver regeneration, recovery of the lungs, kidneys, and spinal cord after injury [25].

The evolution of innate immunity proteins (receptors, adaptors, etc.) of multicellular organisms was studied for more than two decades. The most deeply analyzed family of Toll- and Toll-like receptors (TLRs) demonstrates high level of sequence conservation; this family was traced to the origins of eukaryotic life [202]. Detailed analysis of TLRs and the components of TLR-dependent signaling demonstrated a high conservation of signal-transducing domains from teleost (fish) to mammals, while pathogens recognition domains were more divergent [203]. In addition, high genomic and amino acid conservation was found for NOD1 and NOD2 receptors in all analyzed vertebrate species [204]. Within the framework of our hypothesis, these data indicate a high conservation of the executive mechanisms of the phenoptotic program, at least in the evolution of vertebrates.

Hence, phenoptosis appears to be almost as old as the life itself. In prokaryotes, phenoptosis (equivalent to programmed cell death) could evolve as a defense strategy against phage infection. The innate, as well as adaptive prokaryotic “immune systems” are in close evolutionary and functional relations with the PCD modules [191]. In humans these links are manifested in leading role of innate immunity in the severe course of various viral and bacterial infections.

## 10. Conclusions

The studies considered above show the crucial role of innate immunity in the execution of phenoptosis, using examples from prokaryotes to humans. The lethal manifestations of innate immunity discussed above may not be the result of its aberrant hyperstimulation, but manifestations of an altruistic suicide program that protects the population from the spread of pathogens or dangerous pathologies. The programmed cell death of prokaryotes is in close evolutionary and functional relations with their “immune systems”, which prevent the spread of phage infections. In humans, activation of innate immune responses during severe pathologies initiates and executes programs aimed at cleansing the population of contagious and disabled individuals. Suppressing these atavistic programs with various anti-inflammatory therapies, including the use of mitochondria-targeted antioxidants, very long omega-3 fatty acids, DAMPs antagonists, etc., could contribute to strategies to combat the death program(s), which should be the main goal of future medicine.

## Figures and Tables

**Table 1:** Typical damage-associated molecular patterns (DAMPs).

#	Origin	DAMPs	Receptors	Ref
1	Nucleus	HMGB1	TLR2, TLR4, RAGE	[29]
		CIRP	TLR4, TREM-1	[30]
		Histones	TLR2, TLR4	[47]
		SAP130	Mincle	[26]
		IL-1α	IL1R1	[31]
		IL-33	ST2	[31]
		DNA	cGAS, AIM2, RAGE, IFI16	[21]
2	Cytosol	S100 proteins	TLR2, TLR4, RAGE	[31]
		HSPs	TLR2, TLR4, CD91	[35]
		F-Actin	DNGR-1, TREM1	[32]
		Cyclophilin A	CD147	[33]
		Peroxiredoxin 1	TLR4	[34]
		Oxidized hemoglobin, heme	TLR4	[36]
		Amyloid β	TLR4	[48]
		ATP, ADP	P2X7R, P2Y2R, P2Y12R,	[40]
		Uric acid	TREM-1, TLR2, TLR4, P2X7, NLRP3	[26]
		mRNA	TLR3	[26]
		microRNAs	TLR7	[26]
		SNAPIN	TLR2	[26]
		AGEs	RAGE	[26]
3	Mitochondria	Formyl peptides	FPR1	[43]
		mtDNA	TLR9, NLRP3	[43]
		Cardiolipin	NLRP3, TREM2	[49]
		Cytochrome *c*	TLR4	[44]
		Oxygenated mitochondrial fatty acids	TRL4	[50,51]
		TFAM	RAGE	[43]
4	ER, secretory granules, autophagosomes	Defensins	TLR4	[37]
		Cathelicidins	P2X7, FPR2	[37]
		Eosinophil-derived neurotoxin	TLR2	[38]
		Granulisin	TLR4	[39]
		Calreticulin	CD91	[52]
		Gp96	TLR2, TLR4, CD91	[52]
		Sequestosome-1 (SQSTM1 or p62)	INSR	[53]
5	Extracellular matrix	Heparan sulphate, versican, aggrecan	TLR4	[42]
		Proteoglycans (biglycan, decorin, etc.)	TLR2, TLR4, CD14, NLRP3	[42]
		Tenascin-C	TLR4	[42]
		Fibrinogen	TLR4	[42]
		Fibronectin	TLR2, TLR4	[42]
		Low molecular weight hyaluronan	TLR2, TLR4, NLRP3	[42]
6	Tumor cells	Annexin A1	FPR1	[45]
		PAUF	TLR4	[45]
		API5	TLR4	[45]
		Rps-3	TLR4	[45]

**Abbreviations:** AGEs, advanced glycation end products; AIM2, absent in melanoma 2;API5, apoptosis inhibitor 5; cGAS, cyclic GMP-AMP synthase; CIRP, cold-inducible RNA-binding protein; DNGR1, dendritic cell natural killer lectin group receptor 1; FPR1, Formyl peptide receptor 1; HMGB1, high mobility group box-1 protein; HSPs, heat shock proteins; INSR, insulin receptor; Mincle, macrophage inducible Ca^2+^-dependent lectin receptor; NLRP3, NLR family pyrin domain containing 3; P2X7R, P2X7 receptor; P2Y2R, P2Y2 receptor; PAUF, pancreatic adenocarcinoma up-regulated factor; RAGE, receptor for advanced glycation end products; Rps-3, 40S ribosomal protein S3; SAP130, Sin3A Associated Protein 130; SNAPIN, SNARE-associated protein Snapin; ST2, Suppression of tumorigenicity 2 receptor; TFAM, mitochondrial transcription factor A; TLRs, Toll- like receptors; TREM1, triggering receptors expressed on myeloid cells 1.

## Data Availability

Not applicable.
